# The Relation of Social-ecological Factors and Health Literacy to Medical Students’ Alcohol Use Behavior in Hubei Province, China

**DOI:** 10.34172/jrhs.2023.134

**Published:** 2023-12-29

**Authors:** Meihua Yin, Suneerat Yangyuen, Thidarat Somdee

**Affiliations:** ^1^Faculty of Public Health, Mahasarakham University, Mahasarakham Province, Thailand

**Keywords:** Health literacy, Alcohol drinking, Medical students, Adolescent

## Abstract

**Background:** Health literacy (HL) plays a crucial role in the adolescent’s behavior. Inadequate HL can contribute to engaging in risky alcohol consumption, but little is known about this relationship among medical students. We aimed to investigate the relationship between HL and alcohol use among Chinese medical students.

**Study Design:** A cross-sectional design.

**Methods:** This research was conducted on 1146 medical students in Hubei province, China. The data were collected using a web-based online questionnaire. Multiple logistic regression was applied to investigate factors related to alcohol use.

**Results:** Approximately 45.3% of medical students were drinkers, about 11.5% were hazardous drinkers, and 33.8% were low-risk drinkers; furthermore, about 49.3% of them reported lower levels of HL. In both the low-risk and hazardous drinking groups, the subjects who had low levels of all six dimensions of HL were more likely to use alcohol after adjusting for other covariates, including cognitive skill (adjOR_for low-risk_=3.50; 95% CI: 2.41, 5.07, adjOR_hazardous_=2.07; 95% CI: 1.22, 3.51), access skill (adjOR_for low-risk_=2.11; 95% CI: 1.46, 3.05, adjOR_hazardous_=2.40; 95% CI: 1.37, 4.19), communication skill (adjOR_for low-risk_=1.72; 95% CI: 1.20, 2.47, adjOR_hazardous_=2.21; 95% CI: 1.22, 4.00), self-management skill (adjOR_for low-risk_=1.73; 95% CI: 1.15, 2.59, adjOR_hazardous_=4.01; 95% CI: 1.91, 8.44), media skill (adjOR_for low-risk_=1.50; 95% CI: 1.01, 2.23, adjOR_hazardous_=4.68; 95% CI: 2.15, 10.17), and decision skill (adjOR_for low-risk_=2.12; 95% CI: 1.49, 3.00, adjOR_hazardous_=2.25; 95% CI: 1.35, 3.74).

**Conclusion:** Inadequate HL plays an important role in increasing alcohol use. Thus, prevention and intervention strategies should be based on improving medical students’ HL.

## Background

 Alcohol use has been identified as the health behavior most strongly associated with substance dependence and an increased risk of chronic disease in adulthood.^1^ The World Health Organization (WHO) reports that approximately 26.5% of youth are currently consuming alcohol.^2^ In China, alcohol consumption among medical students is a serious public health problem. In 2012, the rate of alcohol use among adolescents was 21.8%, increasing to 41.2% in 2016^2^ and 48.09% in 2022.^3^ Previous studies have shown that the main risk factors associated with alcohol use among young people include smoking,^4^ monthly household income,^5,6,7^ family environment,^8,9^ intimate partners ^9^, access to alcohol,^10,11^ alcohol expectations,^12,13^ social norms of alcohol^14-16^ and health literacy (HL).^17,18^ In addition, due to inadequate HL, individuals have limited ability to access, understand, interpret, and evaluate drug-related information and low self-management knowledge to make appropriate decisions to prevent or avoid the risk of drinking or to abstain from drinking.^5,19^ Thus, limited HL may lead to alcohol dependence, poor treatment outcomes, and relapse.^20^ However, low HL may influence alcohol use, but no studies have been conducted with medical students in China, and there are no statistics or evidence on this topic. Therefore, investigating the effect of HL on alcohol use among medical students may help reduce the risk of their alcohol behavior and provide guidelines for designing alcohol prevention interventions.

## Methods

###  Study population 

 This cross-sectional study was conducted from October 2022 to May 2023 at four medical colleges in Hubei Province, China. Eligible participants were students aged 17–24 with no communication problems and those who were willing to participate, while those who provided incomplete responses were excluded.

 The calculation of the sample size was conducted using Daniel’s formula.^21^ The percentage of alcohol use among college students (49.92%) was estimated according to Sun et al,^22^ with a 95% confidence interval (CI) and an expected precision of 3%. This accounted for 1031 participants; then in addition to 10% compensation for nonresponse or dropout, the final sample size was 1146. A total of 1402 medical students enrolled, and 256 were excluded because of incomplete questionnaires. Therefore, a multi-stage sampling method was used to select 1146 eligible students. In the first stage, four medical colleges from different orientations were selected according to the geographical distribution of medical specialties in education institutions in Hubei province. In the second stage, the three majors in each college were selected using the lottery method from the list of majors in each college. In the third stage, the medical students were selected by systematic random sampling at each college. Every fourth student on the list was selected as a participant and excluded in the case the student was absent or unwilling to participate; then the student next on the list was taken in. In this study, a socio-ecological model was stimulated that focuses on the interplay among individual, interpersonal, and community-level variables on alcohol behavior.

###  Instruments

 The self-reported questionnaire by web-based online software consists of four parts as follows:

###  Covariates

 Part 1: The individual-level variables contain all socio-demographic factors, alcohol expectancies (AEs), and HL. Socio-demographic factors such as age, gender, monthly household income, and smoking are categorized as dichotomous variables. Moreover, the AE variable, measured by a self-reported questionnaire adapted from Ham et al reflects the expectations of a positive and negative effect of alcohol consumption ^23^. A scoring questionnaire ranging from 1 (disagree) to 4 (agree) consists of 15 items (8 items for positive alcohol expectancies [PAEs] and 7 items for negative alcohol expectancies [NAEs]). The total scores were defined by summing the scores across all items of each dimension (for PAEs, range 8–32 and for NAEs, range 7–28), and (Cronbach’s α was 0.85 and 0.83, respectively). HL was assessed by the Alcohol Health Literacy Scale adapted by Ponrachom,^24^ which reflects an individual’s capacity to change his/her alcohol use behavior. This summed rating scale comprised 36 items across 6 dimensions: cognitive skill, access skill, communication skill, self-management skill, media literacy skill, and decision skill. The total scores were calculated with a summary of the scores of all items (ranging from 30 to 150). We divided each dimension scale into two groups (high and low) based on the median method. The scale showed good internal consistency (Cronbach’s α was 0.83).

 Part 2: The interpersonal-level variables contain two items reflecting the extent to which family and peer members consumed alcohol.

 Part 3: The community-level variables include the social norm of alcohol use and access to alcohol use. We administered the social norm of alcohol use scale developed by Songklang and Yangyuen,^25^ which measures social value regarding alcohol’s harmful effects. This scale consists of 9 items rated on a five-point scale, ranging from 1 (strongly disagree) to 5 (strongly agree). The total scores were calculated by summing the scores of all items (range 9–45), with higher scores indicating greater perceived alcohol harmful effects. We dichotomize this scale (high and low) by median. It demonstrated good internal consistency (Cronbach’s α was 0.87). Access to alcohol use was defined as the respondents asking, “It is easy to buy alcohol in your community if you want to”. This variable was categorized as a dichotomous variable (Y/N).

###  Outcome variable

 Part 4: The primary outcome of this study is alcohol use behavior. The participants were asked whether or not they had ever used alcohol in the past 12 months. We applied the most widely used AUDIT scale in China, which was introduced and translated into Chinese in 1999 by Li et al.^26^ The scale consists of 10 questions: questions 1-3 assess drinking behavior, questions 4-6 assess dependence, and questions 7–10 measure consequences or problems related to drinking. The total score of the scale is 40 points, with a score of 0–7 as low-risk, 8–15 as hazardous use, 16–19 as harmful use, and 20 or above as alcohol dependence. This scale has good internal consistency (Cronbach’s α was 0.85).

###  Statistical analysis

 Descriptive analyses were conducted on the characteristics of all variables. The bivariate odds ratio (OR) was computed to estimate the strength of associations between individual-level, interpersonal-level, and community-level variables and alcohol consumption. The adjusted OR estimated from multiple logistic regression indicated the association between HL and alcohol use after adjustment for all other predictors. We developed a series model such as model 1, only individual-level variables were entered in the model. Then, in model 2, all interpersonal-level variables were entered into model 1. Finally, in model 3, community-level variables were introduced into model 2. The alcohol consumption data for this study were divided into three groups: never drinking, low-risk drinking (AUDIT score 0-7), and hazardous drinking (AUDIT score 8-15). In all models, the reference group for the outcome variable was never drinking. The statistically significant level was set at a *P*-value < 0.05, and all data were analyzed using SPSS version 25.0 (IBM Corp., Armonk, NY, USA).

## Results

 The majority of medical students were female (51.8%), and the median age was 19 years. Approximately 45.3% of medical students used alcohol, about 11.5% were hazardous drinkers, and 33.8% were low-risk drinkers. More than one-third (38.5%) had a monthly household income of 300–5000 CNY, and more than half reported that their family member (59.3%) and peer (51.7%) were drinking. Most medical students reported high levels of PAEs (50.1%) or NAEs (54.7%). Moreover, the overall HL scale scores showed that about 49.3% had a low level of HL. When divided by alcohol use behavior, about 94.7% of hazardous drinkers and 72.9% of low-risk drinkers had a low level of HL (Table 1).

**Table 1:** Distribution of individual, interpersonal, and community-level variables by alcohol use

**Variables**	**Total** **(N=1146)**		**Hazardous** **(n=132)**		**Low-risk** **(n=387)**		**No drinking** **(n=627)**		***P *****value**
	**No.**	**%**	**No.**	**%**	**No.**	**%**	**No.**	**%**	
**Individual-level/ Health literacy**									
Cognitive skill									0.027
Low	326	28.4	49	37.1	171	44.2	106	16.9	
High	820	71.6	83	62.9	216	55.8	521	83.1	
Access skill									0.002
Low	498	43.5	99	75.0	231	59.7	168	26.8	
High	648	56.5	33	25.0	156	40.3	459	73.2	
Communication skill									0.001
Low	554	48.3	109	82.6	248	64.1	197	31.4	
High	592	51.7	23	17.4	139	35.9	430	68.6	
Self-management skill									0.004
Low	560	48.9	120	90.9	257	66.4	183	29.2	
High	586	51.1	12	9.1	130	33.6	444	70.8	
Media skill									0.015
Low	572	49.9	122	92.4	256	66.1	194	30.9	
High	574	50.1	10	7.6	131	33.9	433	69.1	
Decision skill									0.003
Low	515	44.9	83	62.9	226	58.4	206	32.9	
High	631	55.1	49	37.1	161	41.6	421	67.1	
Total health literacy									0.018
Low	565	49.3	125	94.7	282	72.9	158	25.2	
High	581	50.7	7	5.3	105	27.1	469	74.8	
Age (y)									0.004
≥ 19	714	62.3	93	70.5	255	65.9	366	58.4	
< 19	432	37.7	39	29.5	132	34.1	261	41.6	
Gender									0.009
Male	552	48.2	79	59.8	201	51.9	272	43.4	
Female	594	51.8	53	40.2	186	48.1	355	56.6	
Monthlyhousehold income (CNY)									0.108
< 1000	179	15.6	23	17.4	59	15.2	97	15.5	
1000-3000	349	30.5	26	19.7	114	29.5	209	33.3	
3001-5000	441	38.5	43	32.6	135	34.9	263	41.9	
> 5000	177	15.4	40	30.3	79	20.4	58	9.3	
Smoking									0.001
Yes	283	24.7	58	43.9	124	32.0	101	16.1	
No	863	75.3	74	56.1	263	68.0	526	83.9	
Positive alcohol expectancies									0.012
High	574	50.1	85	64.4	240	62.0	249	39.7	
Low	572	49.9	47	35.6	147	38.0	378	63.3	
Negative alcohol expectancies									0.361
High	627	54.7	81	61.4	219	56.6	327	52.2	
Low	519	45.3	51	38.6	168	43.4	300	47.8	
**Interpersonal-level**									
Family member alcohol use									0.001
Yes	680	59.3	99	75.0	260	67.2	321	51.2	
No	466	40.7	33	25.0	127	32.8	306	48.8	
Peer alcohol use									0.005
Yes	593	51.7	85	64.4	244	63.0	264	42.1	
No	553	48.3	47	35.6	143	37.0	363	57.9	
**Community-level**									
Social norm of alcohol use									0.179
High	609	53.1	55	41.7	180	46.5	374	59.6	
Low	537	46.9	77	8.3	207	53.5	253	40.4	
Easy access to alcohol									0.013
Yes	814	71.0	108	81.8	300	77.5	406	64.8	
No	332	29.0	24	18.2	87	22.5	221	35.2	

*Note.* CNY: Chinese Yuan.

###  Bivariate models

 In both the low-risk and hazardous levels of the drinking category, the lower levels of total HL and all 11/17/2023 dimensions of HL were associated with increased odds of alcohol use. Medical students who were male, aged 19 years and older, and smoking had a monthly household income of more than 5000 CNY, family members and peers drinking had a high level of PAEs or social norm of alcohol use, and students with easy access to alcohol were more likely to use alcohol. However, there was no significant association between monthly household income less than 5000 CNY, NAEs, and alcohol use (Tables 2 and 3).

**Table 2:** Odds ratios and 95% confidence intervals from multinomail logistic regression for low-risk drinking

**Variables**	**Bivariate**		**Model 1**		**Model 2**		**Model 3**	
	**Unadjusted OR** **(95% CI)**	***P*** **-value**	**Adjusted OR** **(95%CI)**	***P*** **-value**	**Adjusted OR** **(95%CI)**	***P*** **-value**	**Adjusted OR** **(95%CI)**	***P *** **value**
**Individual-level**								
Health literacy								
Low cognitive skill (ref: High)	3.89 (2.91, 5.19)	0.001	3.31 (2.32,4.74)	0.001	3.48 (2.42, 5.02)	0.001	3.50 (2.41, 5.07)	0.001
Low access skill (ref: High)	4.04 (3.09, 5.29)	0.001	2.07 (1.45,2.97)	0.001	2.08 (1.44, 3.00)	0.001	2.11 (1.46, 3.05)	0.001
Low communication skill (ref: High)	3.89 (2.98, 5.08)	0.001	1.81 (1.27,2.58)	0.001	1.74 (1.21, 2.49)	0.002	1.72 (1.20, 2.47)	0.003
Low self-management skill (ref: High)	4.76 (3.65, 6.29)	0.001	1.89 (1.27,2.81)	0.002	1.79 (1.19, 2.67)	0.005	1.73 (1.15, 2.59)	0.008
Low media skill (ref: High)	4.36 (3.32, 5.71)	0.001	1.48 (1.00,2.19)	0.048	1.54 (1.03, 2.28)	0.032	1.50 (1.01, 2.23)	0.043
Low decision skill (ref: High)	2.86 (2.20, 3.72)	0.001	2.32 (1.65,3.24)	0.001	2.19 (1.55, 3.08)	0.001	2.12 (1.49, 3.00)	0.001
Low total health literacy (ref: High)	7.97 (5.98, 10.62)	0.001	1.73 (1.07,2.80)	0.024	1.76 (1.08, 2.86)	0.021	1.79 (1.10, 2.91)	0.019
Age ≥ 19 (ref: < 19 y)	1.37 (1.05, 1.79)	0.017	1.22 (0.88,1.70)	0.228	1.18 (0.84, 1.65)	0.333	1.16 (0.83, 1.63)	0.368
Male (ref: Female)	1.41 (1.09, 1.81)	0.008	1.58 (1.15, 2.18)	0.005	1.65 (1.19, 2.29)	0.003	1.63 (1.17, 2.27)	0.003
Monthly household income (ref: < 1000 CNY)								
1000-3000	0.89 (0.60, 1.33)	0.590	1.23 (0.75, 2.00)	0.409	1.30 (0.79, 2.14)	0.300	1.26 (0.76, 2.09)	0.357
3001-5000	0.84 (0.57, 1.23)	0.387	1.08 (0.67, 1.75)	0.740	1.16 (0.71, 1.90)	0.531	1.13 (0.69, 1.85)	0.619
> 5000	2.23 (1.40, 3.57)	0.001	2.41 (1.34, 4.35)	0.003	2.53 (1.38, 4.63)	0.003	2.46 (1.33, 4.52)	0.004
Smoking (ref: No)	2.45 (1.81, 3.32)	0.001	1.75 (1.18, 2.58)	0.005	1.73 (1.17, 2.55)	0.006	1.70 (1.14, 2.52)	0.008
High PAEs (ref: Low)	2.47 (1.91, 3.21)	0.001	2.98 (2.09, 4.26)	0.001	2.82 (1.96, 4.05)	0.001	2.84 (1.98, 4.09)	0.001
High NAEs (ref: Low)	1.19 (0.92, 1.54)	0.169	0.80 (0.56,1.14)	0.233	0.77 (0.54, 1.10)	0.153	0.77 (0.53, 1.10)	0.158
**Interpersonal-level**								
Family member alcohol use (ref: No)	1.95 (1.49, 2.54)	0.001	-	-	1.49 (1.07, 2.08)	0.016	1.47 (1.05, 2.05)	0.022
Peer alcohol use (ref: No)	2.34 (1.80, 3.04)	0.001	-	-	1.96 (1.41, 2.73)	0.001	1.95 (1.40, 2.72)	0.001
**Community-level**								
High social norm of alcohol use (ref: Low)	0.58 (0.45, 0.76)	0.001	-	-	-	-	0.91 (0.65,1.26)	0.584
Easy access to alcohol (ref: No)	1.87 (1.40, 2.50)	0.001	-	-	-	-	1.70 (1.18, 2.45)	0.004

*Note.* OR: Odds ratio; CI: Confidence interval; CNY:Chinese Yuan; PAEs: Positive alcohol expectancies; NAEs: Negative alcohol expectancies.

**Table 3:** Odds ratios and 95% confidence intervals from multinomail logistic regression for hazardous drinking

**Variables**	**Bivariate**		**Model 1**		**Model 2**		**Model 3**	
	**Unadjusted OR** **(95%CI)**	***P*** **-value**	**Adjusted OR** **(95%CI)**	***P*** **-value**	**Adjusted OR** **(95%CI)**	***P*** **-value**	**Adjusted OR** **(95%CI)**	***P*** **-value**
**Individual-level**								
Health literacy								
Low cognitive skill (ref: High)	2.90 (1.92, 4.37)	0.001	2.01 (1.20, 3.35)	0.007	2.08 (1.24, 3.50)	0.006	2.07 (1.22, 3.51)	0.006
Low access skill (ref: High)	5.19 (4.32, 7.62)	0.001	2.48 (1.43, 4.28)	0.001	2.43 (1.40, 4.24)	0.002	2.40 (1.37, 4.19)	0.002
Low communication skill (ref: High)	4.34 (3.39, 6.72)	0.001	2.36 (1.32, 4.24)	0.004	2.24 (1.24, 4.04)	0.007	2.21 (1.22, 4.00)	0.009
Low self-management skill (ref: High)	5.26 (4.07, 6.50)	0.001	4.47 (2.14, 9.32)	0.001	4.17 (1.99, 8.75)	0.001	4.01 (1.91, 8.44)	0.001
Low media skill (ref: High)	4.72 (3.98, 5.73)	0.001	4.67 (2.15, 10.12)	0.001	4.84 (2.23, 10.52)	0.001	4.68(2.15, 10.17)	0.001
Low decision skill (ref: High)	3.46 (2.34, 5.11)	0.001	2.51 (1.53, 4.11)	0.001	2.35 (1.42, 3.89)	0.001	2.25 (1.35, 3.74)	0.002
Low total health literacy (ref: High)	7.81 (5.97, 10.78)	0.001	3.22 (1.19, 8.66)	0.021	3.32 (1.23, 8.99)	0.018	3.48 (1.28, 9.45)	0.014
Age ≥ 19 (y) (ref: < 19)	1.70 (1.13, 2.55)	0.010	1.49 (0.90, 2.46)	0.117	1.47 (0.88, 2.45)	0.133	1.48 (0.88, 2.47)	0.131
Male (ref: Female)	1.94 (1.32, 2.85)	0.001	2.24 (1.39, 3.61)	0.001	2.35 (1.45, 3.81)	0.001	2.61 (1.17, 5.81)	0.018
Monthly household income (ref: < 1000 CNY)								
1000-3000	0.52 (0.28, 0.96)	0.038	0.74 (0.35, 1.56)	0.435	0.79 (0.37, 1.68)	0.544	0.76 (0.36, 1.63)	0.495
3001-5000	0.69 (0.39, 1.20)	0.191	0.87 (0.43, 1.75)	0.710	0.94 (0.46, 1.91)	0.878	0.92 (0.45, 1.87)	0.826
> 5000	2.90 (1.58, 5.33)	0.001	2.68 (1.23, 5.84)	0.013	2.69 (1.21, 5.96)	0.014	2.61 (1.17, 5.81)	0.018
Smoking (ref: No)	4.08 (2.72, 6.11)	0.001	2.39 (1.42, 4.03)	0.001	2.37 (1.40, 4.01)	0.001	2.30 (1.35, 3.91)	0.002
High PAEs (ref: Low)	2.74 (1.85, 4.05)	0.001	3.03 (1.79,5.12)	0.001	2.81 (1.65, 4.78)	0.001	2.81 (1.65, 4.80)	0.001
High NAEs (ref: Low)	1.45 (0.99, 2.13)	0.055	0.93 (0.56,1.56)	0.803	0.91 (0.54, 1.52)	0.720	0.91 (0.54,1.53)	0.726
**Interpersonal-level**								
Family member alcohol use (ref: No)	2.86 (1.87, 4.37)	0.001	-	-	1.87 (1.11, 3.16)	0.018	1.88 (1.11, 3.19)	0.019
Peer alcohol use (ref: No)	2.48 (1.68, 3.67)	0.001	-	-	1.81 (1.10, 2.97)	0.018	1.79 (1.09, 2.95)	0.021
**Community-level**								
High social norm of alcohol use (ref: Low)	0.48 (0.33, 0.70)	0.001	-	-	-	-	0.85 (0.52, 1.38)	0.519
Easy access to alcohol (ref: No)	2.45 (1.52, 3.92)	0.001	-	-	-	-	2.09 (1.17, 3.74)	0.012

*Note.* OR: Odds ratio; CI: Confidence interval; CNY: Chinese Yuan; PAEs: Positive alcohol expectancies; NAEs: Negative alcohol expectancies.

 In multinomial logistic analyses, the findings were almost similar for both hazardous and low-risk drinking categories. Model 1 indicated that the greater use of alcohol is associated with lower levels of total HL and six dimensions of HL, being male, monthly household income greater than 5000 CNY, smoking, and a higher level of PAEs. In model 2, interpersonal-level factors were added to model 1, family member and peer alcohol use were significantly related to drinking. In model 3, community-level factors were added to the model. The results revealed a similar association between the individual and interpersonal-level variables and alcohol use in model 2. Furthermore, total HL and six dimensions of HL remained significantly associated with alcohol use after controlling for other predictors. Additionally, students who had easy access to alcohol were more likely to use it (Tables 2 and 3).

## Discussion

 This study showed that medical students with low HL were more likely to drink alcohol, and the results of this study are consistent with those of Yangyuen et al^5^ who reported that adolescents with inadequate HL are more likely to consume alcohol. One explanation is that this phenomenon occurs due to the considerable academic and employment pressures that medical students are currently facing and the tedium of medical knowledge that makes them willing to choose easy access to alcohol for a brief period of pleasure.^27^ In addition, the study by Rolova et al demonstrated a strong link between low HL and alcohol consumption, which implies that in a low HL situation, people often tend to have insufficient access to, understanding of, and assessment of knowledge related to the alcohol harms, as well as the motivation and ability to self-manage, and are prone to alcohol consumption behaviors under such circumstances.^28^

 In addition, the results revealed that other social-ecological factors related to alcohol use such as smoking behavior were also closely related to the occurrence of drinking behavior ^29^, which is consistent with that of Motschman and Tiffany.^30^ This may be because smoking is often used as a means of social interaction, namely, often taking a cigarette from the other party before a conversation and then moving on to the second stage of deepening the relationship through drinking as both parties become more familiar with it, a psychological motivation that, if not strictly controlled by social norms, can inadvertently lead to a negative social culture. The current study also found that easy access to alcohol is an important reason for their drinking.^10,11,31^ Another reason is that there is a lack of proper guidance and strict supervision in the family, school, and society; furthermore, the government’s weak regulation of the marketplace is another reason that leads to arbitrary purchases in supermarkets, retail stores, bars, and the like. With the lack of regulation on both buyers and sellers, obtaining alcohol becomes easier, and drinking behavior increases.^32^ Moreover, behavioral modeling by parents and peers plays a key role. Parents’ drinking behaviors are passed on to their children in their daily lives, which can lead children to believe that drinking is a normal and healthy behavior. Peers also influence adolescents’ receptivity to health information, including alcohol-related information and health decisions through peer pressures and lifestyle practices in their age groups.^8,9,33^ Thus, it may be difficult for them to raise their health awareness and perception of the risks of drinking.^34^ However, this finding is inconsistent with that of Freisthler et al^35^ who reported that under the strict regulatory system in the United States, most parents do not drink alcohol in front of their children and that parents choose meaningful activities to increase their adolescents’ health information and reduce their risk of alcohol exposure.

 Furthermore, this study showed that alcohol consumption is strongly associated with individuals’ expectations of alcohol.^12,13^ PAEs were positively associated with alcohol consumption among medical students, whereas NAEs were negatively associated with alcohol consumption, a finding that is inconsistent with Chisolm et al^36^ One possible explanation is that individuals decide whether to drink alcohol based on their expected positive and negative consequences of alcohol consumption, and that medical students with PAEs may enjoy the euphoric feeling presented by their alcohol-paralyzed brain, a feeling that may cause them to slowly develop an alcohol addiction and depend on alcohol for a moment of pleasure. Consequently, PAEs are thought to promote alcohol use, while NAEs are thought to have the opposite effect.^37^ In addition, AEs can be obtained by observing parental or peer drinking behaviors and learning attitudes toward drinking, which have the most direct impact on adolescents.

 We also found that males are the main group of people who experience hazardous and low-risk drinking, which may still be inextricably linked to traditional Chinese culture. Males have been influenced by traditional Chinese culture and environment during their growth and development, and this finding is consistent with Ghoreishi et al^38^ who showed that males have more freedom in terms of their family and social relationships. As a result, they have more access to alcohol. In addition, alcohol use is strongly linked to family economic status.^5-7^ The amount of family income is related to the status of the pocket money that the adolescents can dispose of as they please, so adolescents with high family income will have more pocket money at their disposal to the extent that they can get alcohol quickly when they want to get it.

 This study has some limitations. First, because of the cross-sectional design, it is not possible to infer temporal and causal relationships. Second, although we used a social-ecological model, there are two dimensions that we did not cover: institutions and public policies because they are currently lacking clarity, so this can be the focus of future research. Third, the data were collected by self-report, which can be implicated in social desirability bias. To minimize self-report bias, validated and standardized instruments were used. Fourth, our subjects were medical students who may have different experiences of alcohol use from other adolescents who were non-medical students and non-academic youth and were in communities; thus, caution must be used when generalizing the results to other groups. For example, the study by Chi et al^39^ reported that the alcohol consumption of adolescents aged 18–20 years who resided in six Chinese cities is 31.8%, and the study by Chen et al showed that the drinking rate of medical students is 64.1%, and that of non-medical students is 73.2%.^40^

 Despite these limitations, our study has a compensatory strength that allows for large sample sizes and controls for a wide range of covariates. The results provided evidence of risk factors for alcohol use, and low HL is an important contributor to alcohol use among medical students. Further, randomized controlled trials are needed to verify that improving HL is effective in reducing alcohol use, and more effective interventions to reduce alcohol use should be explicitly considered in the design based on the HL model.

**Highlights**

- In China, approximately a quarter of medical students reported low-risk to hazardous alcohol use in the past years.
- Alcohol consumption is higher among medical students with low health literacy.
- Social-ecological factors were associated with both low-risk and hazardous alcohol use among medical students.

## Conclusion

 This study indicated that the three-level factors of a socio-ecological model such as individual-level variables (all six dimensions of HL, low total HL, male, monthly household income > 5000 CNY, smoking, and high PAEs), interpersonal-level variables (family member alcohol use and peer alcohol use), and community-level variables (easy access to alcohol) are related to alcohol use among medical students. Additionally, these results support the idea that improving alcohol HL should be considered a part of the development of an alcohol use reduction program.

## Acknowledgements

 We are grateful to the Faculty of Public Health, Mahasarakham University for research support funding, and we would like to sincerely thank all the study participants for their participation.

## Authors’ Contribution

**Conceptualization: **Suneerat Yangyuen, Meihua Yin, Thidarat Somdee.

**Data curation:** Suneerat Yangyuen, Meihua Yin.

**Formal analysis:** Suneerat Yangyuen, Meihua Yin.

**Funding acquisition:** Suneerat Yangyuen.

**Investigation:** Meihua Yin, Thidarat Somdee.

**Methodology:** Suneerat Yangyuen, Meihua Yin.

**Project administration:** Meihua Yin.

**Resources:** Meihua Yin, Thidarat Somdee.

**Software:** Meihua Yin, Thidarat Somdee.

**Supervision:** Suneerat Yangyuen.

**Validation:** Suneerat Yangyuen, Meihua Yin, Thidarat Somdee.

**Visualization:** Meihua Yin, Thidarat Somdee.

**Writing–original draft:** Suneerat Yangyuen, Meihua Yin, Thidarat Somdee.

**Writing–review & editing:** Suneerat Yangyuen, Meihua Yin, Thidarat Somdee.

## Competing Interests

 There is no conflict of interests.

## Ethical Approval

 Written informed consent was obtained from each subject based on the study information, conducted in accordance with ethical principles, and approved by the Ethics Review Board of Mahasarakham University (Ethics number: 164-053/2566).

## Funding

 We received grant support from the Faculty of Public Health, Mahasarakham University.
